# Role of adrenergic receptors in shock

**DOI:** 10.3389/fphys.2023.1094591

**Published:** 2023-01-16

**Authors:** Mathew Geevarghese, Krishna Patel, Anil Gulati, Amaresh K. Ranjan

**Affiliations:** ^1^ Midwestern University Chicago College of Osteopathic Medicine, Downers Grove, IL, United States; ^2^ Pharmazz Inc., Research and Development, Willowbrook, IL, United States; ^3^ Department of Bioengineering, The University of Illinois at Chicago, Chicago, IL, United States; ^4^ Midwestern University College of Pharmacy Downers Grove, Downers Grove, IL, United States

**Keywords:** adrenergic receptors, centhaquine, hemorrhagic, shock, blood pressure, perfusion, resuscitation, hypovolemia

## Abstract

Shock is a severe, life-threatening medical condition with a high mortality rate worldwide. All four major categories of shock (along with their various subtypes)—hypovolemic, distributive, cardiogenic, and obstructive, involve a dramatic mismatch between oxygen supply and demand, and share standard features of decreased cardiac output, reduced blood pressure, and overall hypoperfusion. Immediate and appropriate intervention is required regardless of shock type, as a delay can result in cellular dysfunction, irreversible multiple organ failure, and death. Studies have shown that dysfunction and downregulation of adrenergic receptors (ARs) are often implicated in these shock conditions; for example, their density is shown to be decreased in hypovolemic and cardiogenic shock, while their reduced signaling in the brain and vasculature decrease blood perfusion and oxygen supply. There are two main categories of ARs, α, and β, each with its subtypes and distributions. Our group has demonstrated that a dose of .02 mg/kg body wt of centhaquine (CQ) specifically activates α2B ARs on venous circulation along with the central α2A ARs after hypovolemic/hemorrhagic shock. Activating these receptors by CQ increases cardiac output (CO) and reduces systemic vascular resistance (SVR), with a net increase in blood pressure and tissue perfusion. The clinical trials of CQ conducted by Pharmazz Inc. in India have demonstrated significantly improved survival in shock patients. CQ improved blood pressure and shock index, indicating better blood circulation, and reduced lactate levels in the blood compared to in-use standard resuscitative agents. After successful clinical trials, CQ is being marketed as a drug (Lyfaquin^®^) for hypovolemic/hemorrhagic shock in India, and United States FDA has approved the phase III IND application. It is anticipated that the phase III trial in the United States will begin in 2023. Thus, we have demonstrated that α2 ARs could be suitable targets for treating or managing hypovolemic/hemorrhagic shock. Further understanding of ARs in shock would help find new potential pharmacological targets.

## 1. Introduction

Shock is the clinical manifestation of extreme dysoxia. Dysoxia is a lack of oxygen supply to, or lack of oxygen used by cells, which limits energy production and leads to cellular dysfunction. Prolonged and/or severe dysoxia can result in an imbalance between oxygen supply and demand at the organ and organ system level, which, if left unchecked or corrected in an untimely or inappropriate manner, causes multiple organ dysfunction and multiple organ failure (MOF) (Roberts, 2016). While all shock types can lead to MOF, differences in their pathophysiology and pathogenesis make it worthwhile to understand the different classifications, especially since different kinds of shock require different management and therapy. It is important to note that these classifications are not meant to be exclusive or binding and that some degree of overlap exists among the different categories. The four significant varieties of shock are hypovolemic—relating to the blood and fluid compartment; distributive—relating to the vasculature; cardiogenic—relating to the heart; and obstructive—relating to the circulatory system (Standl et al., 2018). Briefly, hypovolemic shock is characterized by inadequate perfusion due to a reduction in intravascular volume due to a significant fluid loss, with or without blood loss, and varying degrees of soft tissue injury. Based on their onset, they are categorized into traumatic hemorrhagic or hemorrhagic, traumatic hypovolemic, or hypovolemic subtypes (Bonanno, 2011a; Standl et al., 2018; Spahn et al., 2019). Distributive shock is a state of dysoxia following pathological redistribution of intravascular volume, differing from hypovolemic shock in that volume is not “lost” but rearranged. This can present as volume shifted within the vasculature due to impaired or dysregulated vascular tone and/or volume shifted to the interstitium due to increased vascular permeability. Within the broad category of distributive shock are three subtypes—anaphylactic shock, septic shock, and neurogenic shock (Standl et al., 2018). Cardiogenic shock is a state of hypoperfusion that directly results from the ineffective pump action of the heart. This condition is similar to distributive shock in that there is no actual loss of blood volume either externally or internally. This often presents as decreased blood pressure initially, leading to the body attempting to correct this by contracting major vasculature to raise blood pressure to bring itself to a state of compensated shock (Vahdatpour et al., 2019). The most common source of cardiogenic shock can be directly attributed to the weakening or outright failure of the left ventricle’s ability to pump blood effectively (Hendy and Bubenek-Turconi, 2016). The cause of cardiogenic shock includes various heart diseases, e.g., congestive heart failure and heart attack, which can directly affect contractile strength. Other direct contributors to cardiogenic shock are beta blockers and calcium channel blockers which are negatively inotropic (Haseer Koya and Paul, 2022). Obstructive shock is the final form of shock and is similar to cardiogenic shock in that direct loss of blood volume is not the primary cause. The distinction between this form of shock and cardiogenic shock is that the blood vessels are neither dilated nor contracted, and the heart can pump effectively (no weakening of contractility); instead, it is a physical obstruction within the vessels that prevents proper blood flow, thereby leading to systemic hypoperfusion (Vincent and De Backer, 2013; Wilson et al., 2015). The most common causes of obstructive shock are tension pneumothorax, cardiac tamponade, and pulmonary embolism. Tension pneumothorax is a condition by which one of the lungs collapses, which puts pressure on the pulmonary vasculature. Overall, the common factor in all types of shock is abnormal and inadequate perfusion, as well as insufficient oxygenation and nutrient supply to tissue, which is regulated by the dynamic balance of blood pressure and vascular resistance. The mean arterial pressure (MAP) is akin to blood pressure (BP) and is determined by cardiac output (CO) and total peripheral resistance (TPR) or systemic vascular resistance (SVR). CO is defined as the amount of blood ejected by the left ventricle in 1 minute and can therefore be expressed as CO = stroke volume (SV) x heart rate (HR). At the same time, TPR is the resistance to blood flow in the vasculature and is mainly determined by arterioles which are primary resistance vessels. Therefore, mathematically, MAP can be represented as

MAP=CO x TPR=SV x HR x TPR.

The autonomic nervous system (ANS) is known to reflexly regulate MAP through efferent (sympathetic and or parasympathetic) nerves, which affect cardiac output, vasoconstriction, or vasodilation to change the SVR or TPR. The activity of these nerves is mediated through various catecholamines and their adrenergic receptors (ARs). Catecholamines and ARs work through at least four potential mechanisms. First, AR signaling affects blood pressure through actions on the central nervous system; second, by directly controlling the cardiovascular tone; third, by modulating renal sodium flux; fourth, by affecting renin release and modulating the renin-angiotensin-aldosterone system (Raymond et al., 1990). The catecholamines and various synthetic agonists of ARs are known to initiate these actions by specifically interacting with the receptors. The ARs are linked to guanine nucleotide regulatory proteins, or “G proteins,” which act as intermediary signal-transducing proteins and lead to the generation of intracellular second messengers to regulate the above-mentioned physiological responses (Dohlman et al., 1987). They are integral membrane proteins belonging to the G protein-coupled receptors (GPCR) superfamily and are also known as catecholamine receptors. The ARs are among the most studied GPCRs and belong to the rhodopsin family/class A GPCRs and aminergic receptor subfamily (Wu et al., 2021; Yang et al., 2021). The aminergic receptor subfamily has 42 known members (Kooistra et al., 2013). Bioactive amines act as endogenous agonists. The monoamine neurotransmitters epinephrine and norepinephrine are the endogenous ligands for ARs. Other biogenic monoamines include dopamine, serotonin, and histamine. These monoamines contain an amino group connected to an aromatic ring by a two-carbon chain. However, among them, dopamine is most similar to epinephrine and norepinephrine in chemical structure, each containing a catechol (1,2-dihydroxybenzene) group and, thus collectively, are referred to as catecholamines (Goldstein and Cheshire, 2018). These catecholamines are products of three successive steps in the tyrosine metabolic pathway (Daubner et al., 2011). Besides adrenergic types, the aminergic GPCRs subfamilies include muscarinic, dopaminergic, histaminergic, serotoninergic, and trace amine receptors (Michino et al., 2015). While each subfamily is categorized into subgroups of closely related subtypes, structurally, they have typical GPCRs characteristics, including seven stretches of hydrophobic regions or transmembrane (TM) helices, an extracellular amino terminus, three extracellular loops, three intracellular loops and an intracellular carboxy terminus (Strosberg, 1993). These receptors have several similarities, including one or more extracellular N-linked glycosylation sites near the amino terminus and intracellular regulatory phosphorylation sites near the carboxy terminus. Moreover, the transmembrane domains of these receptors have considerable amino acid sequence homology and form a ligand-binding pocket, which is stabilized by the disulfide bonds among the cysteine residues present in the extracellular domains (Raymond et al., 1990). The cytoplasmic domains contain interacting regions for various G proteins and kinases, which act as second messengers to activate a cascade of events downstream. These events include the activation of specific kinases and subsequent phosphorylation of proteins [e.g., protein kinase A (PKA), protein kinase C (PKC)], the release of intracellular Ca2+ stores, activation of ion channels/pumps, arachidonic acid release, and gene transcription (Raymond et al., 1990). This way, participating second messenger proteins amplify the extracellular signal for adrenergic receptors even by modulating the expression of genes related to different types of cell response. This multilevel regulation of signaling pathways by adrenergic receptors following catecholamines (epinephrine/norepinephrine) binding makes them one of the essential GPCRs in our body, which mediate a large variety of functions that are crucial for our survival (Wu et al., 2021). These functions include vasodilation, vasoconstriction, systolic and diastolic blood pressure, metabolism, and proliferation. Since these essential functions require fine-tuning to maintain homeostasis, various subtypes of adrenergic receptors and their linked effector systems would help regulate these wide arrays of physiological responses. Based on the pharmacology and affinity to different drugs, synthetic agonists, and antagonists for adrenergic receptors, they are classified into three groups: α1, α2, and β. The α1ARs are known to be selectively pharmacologically activated by phenylephrine, α2ARs can selectively be activated by clonidine, while βARs are non-selectively activated by isoproterenol. Each of these adrenergic groups has three subfamilies or subtypes. α1ARs consist of α1A, α1B, and α1D, α2ARs comprise of α2A, α2B, and α2C, and βARs has β1, β2, and β3 subfamily, in humans (Wu et al., 2021). Notably, some species other than humans have a fourth subtype for α2ARs as α2D (Brede et al., 2004). The most common feature of all these subtypes is the recognition of the catechol hydroxy groups present in catecholamines by two serine residues (S5.42 and S5.46) in their TM5 and also conserved in dopamine receptors (Wu et al., 2021). These two positions are non-conserved in other aminergic receptors. However, all aminergic receptors have a common catecholamine binding characteristic with the π-π interaction of the catecholamine aromatic ring to F6.51 and F6.52 of receptors (Wu et al., 2021). Besides the conserved ligand-binding regions of ARs, some non-conserved residues from the ligand-binding pocket are discovered that play a key role in partial agonism and biased AR signaling (Qu et al., 2019). Moreover, it has been shown that ARs differ in their specificity for various ligands and in coupling to G proteins and, thereby, different second messenger systems. For example, activation of β1 or β2ARs subtype stimulates the enzyme adenylyl cyclase and leads to the generation of cyclic adenosine monophosphate (cAMP) mediated by the Gs protein. While activation of α2-ARs causes inhibition of adenylyl cyclase *via* Gi protein. On the other hand, α1-ARs activation stimulates phospholipase C to generate diacylglycerol and inositol trisphosphate (IP3) mediated by a pertussis toxin-insensitive G protein termed Gp (also termed as Gx or Gz) (Wu et al., 2021). Moreover, the tissue-specific distribution of AR subfamilies also helps regulate adrenergic signaling and function. A particular subfamily of ARs is often expressed in different regions of the brain and/or peripheral tissues (e.g., heart and blood vessels) and potentially gets coupled to divergent intracellular/extracellular ligands. α1-ARs are mainly expressed in vascular smooth muscle cells (SMCs), endothelial cells, cardiomyocytes, prostate SMCs and the brain. α2-ARs are present in the autonomic ganglia, sympathetic neurons, central nervous system, pancreas, platelets, kidneys tubular epithelium, vascular SMCs, and gastrointestinal SMCs. β1-ARs exist in cardiomyocytes, kidney SMCs, and adipocytes. β2-ARs are expressed in vascular SMCs, endothelial cells, gastrointestinal SMCs, lung SMCs, cardiomyocytes, uterus SMCs, bladder SMCs, adipocytes, pancreas, eyes (ciliary epithelium), liver, and skeletal muscle. β3-ARs are seen in cardiomyocytes, endothelial cells, adipocytes, brown adipocytes, bladder SMCs, gallbladder, retina, and epithelial cells (Wu et al., 2021). Therefore, selective targeting of a particular AR subtype is critical for elucidating the pathophysiological mechanism involved in various diseases, which may also lead to novel drug discovery and/or better drug design and development.

## 2. Targeting adrenergic receptors in shock

The main role of adrenergic receptors is to facilitate the necessary physiologic changes to body systems to promote survival in times of “crisis.” This crisis can be physiological or pathophysiological, as the compensation in times of shock illustrates. As previously discussed, α-adrenergic receptors play an important role in sympathetic response and vasoconstriction, leading to increased effective circulating volume (ECV) (Reid, 1986) while also decreasing renal blood flow, producing a compensatory activation of the renin-angiotensin-aldosterone system, or RAAS, to further increase effective circulating volume. In conjunction, β-ARs increase the inotropy and chronotropy of the heart and increase plasma renin levels to increase ECV further and strengthen CO and contractility (Yoo et al., 2009; de Lucia et al., 2018).

In times of shock, there is a lack of oxygen, that is, present or dysoxia. As this occurs, the body attempts to compensate by stimulating ARs through α and β stimulation to increase oxygen delivery to tissues, specifically the vital tissues of the brain and heart. Through α ARs stimulation, there is a compensatory increase in blood pressure and effective circulating volume; through β stimulation, there is a compensatory increase in heart rate and contractility. Concomitantly, RAAS activation through decreased renal perfusion mediated by vasoconstriction caused by α receptors, which promotes sodium and water retention, further increasing ECV in an attempt to shunt more oxygen to the brain and heart. As this compensation continues, other organs are starved of nutrients and oxygen, leading to local arteriolar dilation to increase flow to those areas. This flow is postulated to indicate endothelial dysfunction in the brain that initiates the process of decompensated shock (Bonanno, 2011b). The proposed mechanism for this presented by Sharma et al. (2002) states that in decompensatory mechanisms, the role of autonomics is suppressed and the release of endothelin-1 in brain tissue leads to constriction of those vessels, thereby decreasing brain perfusion. In septic shock, it has been proven that the release of pro-inflammatory cytokines and excess nitric oxide (NO) results in the downregulation of adrenergic receptors, including β1, which has been hypothesized to contribute to the overall decreased cardiovascular response in septic shock (de Montmollin et al., 2009). Studies have shown that β-blockers are useful in reducing septic shock-related complications (Balik et al., 2012; Morelli et al., 2013; Du et al., 2016; Shang et al., 2016; Fuchs et al., 2017; Gadallah et al., 2020) in patients with tachycardia. Initial use of β-blockers in septic patients was reported in 1970 when Berk and others used propranolol along with several therapeutic agents for sepsis (e.g., glucagons, high doses steroids, and digitalis) to treat septic patients (*n* = 5), who were suffering from prolonged and therapy-refractory shock before they started this treatment. Three out of five patients showed an increase in blood pressure and recovered, while two died (Berk et al., 1970). In 2006, Gore and Wolfe treated six normotensive septic patients with an infusion of another β-blocker, esmolol (a short-acting β-1 antagonist). The infusion helped in reducing the heart rate by approximately 20%, which decreased cardiac output accordingly, while it did not affect/limit oxygen use, whole-body energy expenditure, or ATP availability (Gore and Wolfe, 2006). Schmittinger et al. (2008), treated 40 septic shock patients with metoprolol through an enteral route along with fluids including noradrenaline, milrinone, and vasopressin. Metoprolol was administered within 48 h after the onset of shock. The results were encouraging as the HR targets between 65 and 95 beats/min were achieved in 39 of 40 patients. Patients had increased SV and stable cardiac indices. Overall, MAP was increased, and noradrenaline and milrinone requirements decreased in most patients; however, in some patients, their doses had to be increased. Clinical studies by Balik et al. (2012); Morelli et al. (2013) have shown that beta-adrenergic blockade using esmolol in patients who had high heart rates even after standard fluid resuscitation caused improvements in cardiovascular performance, including heart rate, left ventricle stroke volume, with no adverse effects. Du et al. (2016) performed esmolol therapy in 63 septic shock ICU patients and observed improved SV and reduced lactate levels than before esmolol therapy, whereas no significant change in blood pressure was observed. A randomized control study of 151 patients with severe sepsis conducted by Shang et al. (2016) showed that IV infusion of esmolol reduced heart rates and the duration of mechanical ventilation in patients treated with esmolol (*n* = 75) compared to patients treated with standard antiseptic shock measures (*n* = 76). Moreover, the esmolol-treated patients had no hazardous effect on circulatory function or perfusion. Fuchs et al. (2017) carried out a single-center trial on adult patients with severe sepsis or septic shock and compared mortality rates between patients who continued and discontinued chronic beta-blocker therapy. The chronic oral beta-blocker treatment included atenolol, bisoprolol, metoprolol, nebivolol, talinolol, carvedilol, propranolol, or sotalol. The study showed decreased 28-day and 90-day mortality rates in patients with the continuation of beta-blockers compared to patients with their discontinuation. Recently, Gadallah et al. (2020) conducted a randomized controlled study on 60 ICU patients with sepsis and observed the beneficial effects of IV infusion of esmolol in addition to the SOC for sepsis on hemodynamics, ICU stay, and mortality. They observed a significant heart rate reduction, decrease in 28-day mortality, and ICU stay in patients who received esmolol than in patients who received only SOC. Although these results indicate that inhibition of β-adrenergic signaling could be useful for treating septic shock, some questions remained unanswered, e.g., how treatment with β-blockers provides benefits? Could it be because the β-adrenergic system is a powerful regulator of the immune system? Hence, further understanding of a highly complexed relationship among inflammatory response, β-ARs and cardiovascular activity following septic shock is required by questioning the ubiquitous nature of the β-adrenergic system and discovering other unknown mechanisms of β-blockers, which may exert their influence in shock.

Apart from β-ARs, α1ARs are downregulated due to pro-inflammatory cytokine release during circulatory failure following shock (Carrara et al., 2021). While in hemorrhagic shock, circulating epinephrine and norepinephrine (catecholamines) fail to maintain vasoconstriction leading to decompensated hypotension, as well as the nitric oxide synthase (NOS) system through the release of endothelin by hypoxia-damaged vasculature (Bonanno, 2011a). In hypovolemic or hemorrhagic shock, the initial cardiovascular imbalance is a fall in the blood volume and compromised vascular capacity that leads to decreased cardiac output and MAP. The fall in MAP and changes in blood chemistry cause withdrawal of the normal inhibitory tone from the cardiovascular control centers in the central nervous system (Bond and Johnson, 1985). Withdrawal of the inhibitory tone results in increased sympathetic activity and stimulation of peripheral adrenergic nerves and the adrenal medulla, which produce nor-epinephrine and epinephrine, respectively. The net result of the interaction of these catecholamines on the peripheral vascular adrenoreceptors determines the magnitude of the compensatory vasoconstriction (Bond and Johnson, 1985). It has been shown that the main adrenoreceptors involved in this compensatory vasoconstriction are presynaptic α2, innervated postsynaptic α1, and extrasynaptic α2 ARs (Bond and Johnson, 1985). The action of epinephrine on α2 ARs and epinephrine, as well as norepinephrine on postsynaptic α1, initiate the compensatory vasoconstriction. However, the action of norepinephrine on the presynaptic α2 inhibits further release of norepinephrine from peripheral nerve terminals (a negative feedback loop) (Langer, 1980), which reduces the effect of postsynaptic α1ARs as well as of extrasynaptic α2 ARs. This autoinhibition of norepinephrine release is thought to be responsible for vascular decompensation following hemorrhage. Therefore, one of the factors determining survival after hypovolemic or hemorrhagic shock is related to the relative dominance of the activity of postsynaptic α1 and extrasynaptic α2 ARs during the compensatory response to the shock (Bond and Johnson, 1985). Thus, these observations indicated that selective agonists of postsynaptic α1 and extrasynaptic α2 ARs could be developed as effective therapeutics for shock. Nonetheless, deeper understanding of the pathophysiological events involved in the decompensatory phase of shock in the context of ARs is required. The decompensatory phase of various types of shock affects the function of the essential organs, e.g., the heart, kidneys, liver, spleen, lungs, and brain. Among all these organs, dysfunction/damage to the heart affects the systemic blood circulation mediated through peripheral macro-circulatory and micro-circulatory systems and disturbs oxygen supply. In normal condition, both systems are interrelated and are mainly systemically regulated by the sympathetic and parasympathetic nervous systems. However, in shock conditions the crosstalk and regulation of macro- and micro-circulation is disrupted (Yeh and Chiu, 2019). The sympathetic nervous system takes over the macro-circulatory system and diverts most of the blood towards coronary and cerebral tissues, and regulation of micro-circulation becomes more local and primarily depends upon the vascular tone (Bonanno, 2011a). The lack of oxygen and the production of inflammatory factors during shock also cause damage to vascular endothelial and smooth muscle cells, which leads to dysregulated vascular tone, and increased vascular dilation and permeability (Bonanno, 2011a). Although the actual cause of vasodilation in shock has not been completely understood, the role of angiopoietin-tie (Ang-Tie) and iNOS signaling in vascular endothelial and smooth muscle cells has been shown (Leligdowicz et al., 2018). Dilation in venous circulation during shock causes increased blood pooling in the venous system and reduces blood return to the heart. This, in turn, affects the stroke volume and cardiac output, ultimately leading to collapsed circulation with low arterial and pulse pressure, low cardiac output, diminished tissue perfusion, and hypothermia (Bonanno, 2011a). In this condition, shock-mediated vasodilatory signaling on smooth muscle cells blunts the effect of vasoconstrictive neurohormones and also makes euvolemic fluid resuscitation ineffective. Therefore, immediate medical interventions are necessary to delay or revert the lethal hypotensive shock effect. Currently, catecholamines, e.g., dopamine, epinephrine, and norepinephrine, are commonly used as hypertensive agents in the resuscitative solution to treat various types of shock (Myburgh, 2006; Myburgh, 2007). These catecholamines regulate blood pressure by binding to the α-adrenergic receptors present in blood vessels. Studies have shown that they have better affinity to α2-than α1 -adrenergic receptors (Pecquery and Giudicelli, 1982; Perez, 2021). Moreover, the α2 subtype genes knock-out study has shown that their vasopressor activity is mediated probably through α2A as the effect of norepinephrine on arterial contraction was abrogated in α2A^−^ mouse but not in α2B^−^ or α2C^−^ mouse (Link et al., 1995; Link et al., 1996; Altman et al., 1999; Philipp et al., 2002). Moreover, studies involving pharmacological antagonists indicated that α2A played major role in regulating release of norepinephrine from presynaptic sympathetic neurons as part of a feedback loop (Trendelenburg et al., 1997). Also, studies in mice lacking the α2A, presynaptic feedback regulation was found severely impaired; however, did not abolish (Altman et al., 1999; Hein et al., 1999). Besides that, the α2C was found to function as an additional presynaptic regulator in all examined central and peripheral nervous tissues, with α2C being more prominent in sympathetic nerve endings than in central adrenergic neurons (Altman et al., 1999; Hein et al., 1999; Trendelenburg et al., 1999; Bucheler et al., 2002). Thus, the arrangement of ARs in presynaptic, postsynaptic and extrasynaptic regions seems to be α2A/α2C presynaptically, α1 postsynaptically, and α2B extrasynaptically. The binding of agonist or epinephrine to α2B induces contraction in the blood vessels as a sympathetic response, while binding of same agonist to α2A/α2C induces release of neurotransmitters (e.g., norepinephrine, serotonin, etc) to postsynaptic α1, and together they induce contraction in arteries as a sympathetic response. However, activation of presynaptic α2A/α2C starts a negative feedback loop later and inhibits the secretion of neurotransmitters required for activation of postsynaptic α1; thus, it curtails the sympathetic response and reduces arterial contraction. Nonetheless, activation of extrasynaptic α2B seems to be independent of this feedback loop because nonselective activation of α2 *via* intravenous agonists injection usually results in a biphasic blood pressure response (Philipp et al., 2002). This could be due to α2B mediated short, hypertensive phase and α2A/α2C mediated long-lasting phase of hypotension. Hence, α2B could be an effective target for inducing contractions in blood vessels during decompensation following shock.

## 3. Development of centhaquine, an α-2 agonist, as a resuscitative agent for hypovolemic/hemorrhagic shock

**FIGURE 1:**
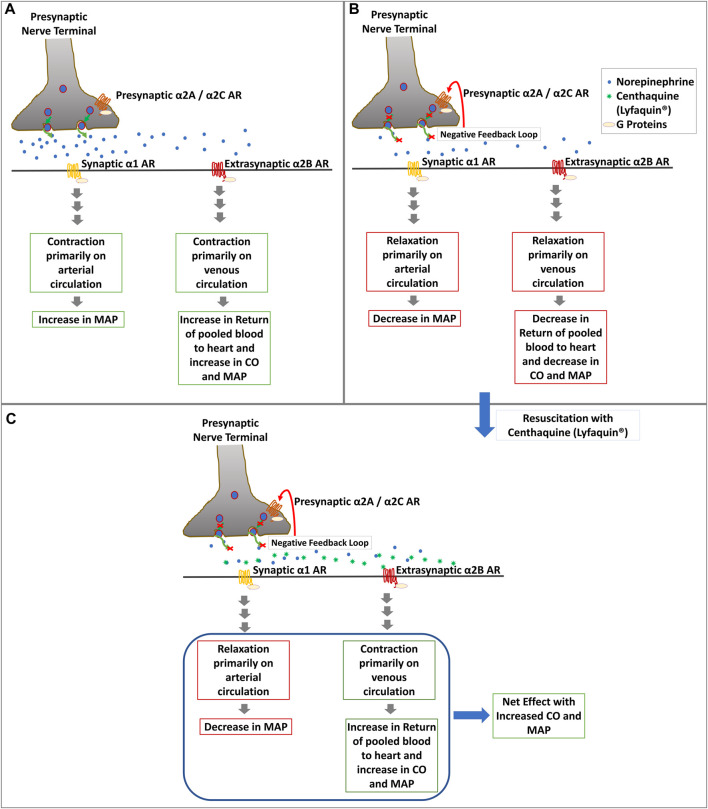
Diagrammatic representation of the potential mechanism of action of CQ (Lyfaquin^®^) resuscitation. **(A)**. Increase in blood pressure in the compensatory phase after shock most likely involves activation of presynaptic α2A/α2C by epinephrine, which leads to the secretion of norepinephrine along with other neurotransmitters (not shown in the figure) from the presynaptic nerve terminal to postsynaptic nerve at the synapse and extrasynaptic tissue. α1 ARs on postsynaptic nerves start downstream sympathetic signaling leading to contraction primarily on the arterial circulatory system and increasing MAP following activation with norepinephrine. Extrasynaptic α2B ARs present primarily in the venous circulatory system also start contraction there and increase CO, which leads to an increase in MAP. However, the inherent feedback loop of norepinephrine and α2A/α2C leads to the decompensatory phase in shock (shown in panel B). **(B)**. Inhibition of α2A/α2C by norepinephrine following the negative feedback loop reduces the secretion of norepinephrine in the synapse, which abrogates the effects described in panel A and leads to relaxation in blood vessels which causes reduced CO and MAP. **(C)**. Resuscitation with CQ (Lyfaquin^®^) helps prevent decompensation or restore the compensatory-like phase, with a net result of increased MAP. CQ is known to preferentially activate α2B ARs, which increases the contraction of the venous circulatory system leading to an increase in the return of pooled blood to the heart and thus increasing the CO and MAP. CQ is also known to activate the central α2A receptors, which would help reduce the SVR and increase tissue perfusion (not shown in this figure).

Distribution of α2A and α2B ARs varies based on the vascular bed and size of the vessels and species (Chruscinski et al., 2001; Philipp et al., 2002; Schwinn and Roehrborn, 2008). Large arteries like the aorta have a high amount of α2A, whereas α2B is abundant in peripheral veins and scarcely in arteries, contributing to most venoconstriction. The dose range of 0.015–0.02 mg/kg body weight centhaquine (CQ) acts on the α2B ARs and induces constriction in most of the veins along with small arteries, which helps in the return of venous pooled blood to the heart (Gulati et al., 2013a; Gulati et al., 2012a; Kontouli et al., 2019; Lavhale et al., 2013; Leonard et al., 2016; O'Donnell et al., 2016a; O'Donnell et al., 2016b; Papapanagiotou et al., 2016; Reniguntala et al., 2015; Gulati et al., 2020). It leads to a significant improvement in CO and MAP after resuscitation (Figure 1). It also acts on the α2A ARs in the brain to produce sympathetic actions, causes dilation of arteries, and reduces the SVR (Bhalla et al., 2013) along with increasing tissue perfusion. Moreover, CQ has shown no significant binding to β-adrenergic receptors, and thus chances of producing arrhythmia are meager. This is a novel mechanism of blood pressure regulation in shock, and no other in-use vasoactive compounds have shown similar effects till now. We have also demonstrated CQ as a highly effective resuscitative agent for the treatment of hypovolemic shock in preclinical and clinical settings (Gulati et al., 2012b; Lavhale et al., 2013; Papapanagiotou et al., 2016; Gulati et al., 2019). Preclinical studies on CQ and hemorrhagic shock have demonstrated the superior effectiveness of CQ compared to commonly used resuscitative agents in reducing lactate level and mortality with increasing cardiovascular activities in animal models following hypovolemic shock (Gulati et al., 2020). In hemorrhagic rats, CQ resuscitation reduced the dependency on norepinephrine needed to maintain a target mean arterial pressure and reduced lactate levels in blood an hour after resuscitation. Blood lactate levels in CQ rats were at 1.65 mmol/L, vs. 4.10 mmol/L in control rats (Gulati et al., 2012b). In another study, rats with hemorrhagic shock dosed with CQ had 44% lower blood lactate levels than control rats, and their survival was increased (Gulati et al., 2013a). This is significant, as shock episodes are known to increase blood lactate levels, which is better correlated with poor patient outcomes and high mortality risk than DO2/VO2 levels (Bakker et al., 1991). We also performed preclinical testing of CQ in a rabbit and a pig blood loss model. We observed rabbits in the control group needed 207.82 ± 9.08 ml, whereas CQ rabbits needed only 133.60 ± 11.91 ml of fluid to maintain the target MAP (Gulati et al., 2013b). Similarly, CQ pigs needed a significantly reduced amount of fluid to maintain target MAP than control pigs. Moreover, CQ pigs achieved target MAP more quickly (7.1 min vs. 36.88 min) than control pigs and had better survival rates; 10/10 in the CQ group vs. 3/10 in the control group, 24 h post resuscitation (Papapanagiotou et al., 2016). Clinical phase I study (NCT02408731) demonstrated its safety and tolerability in humans. Phase II (NCT04056065) and phase III (NCT04045327) clinical trials demonstrated its superior efficacy and effectiveness over currently used resuscitative agents in treating hypovolemic/hemorrhagic shock. The trials were prospective, multicentric, randomized studies conducted in hypovolemic shock patients having systolic blood pressure (SBP) of ≤90 mm Hg and blood lactate levels of ≥2 mmol/L. In the phase III, 71 patients were assigned to the CQ group and 34 patients to the control (saline) group. CQ patients received .01 mg/kg of CQ in 100 ml of normal saline and infused over 1 h along with SOC, while in control patients, 100 ml of only saline along with SOC was infused. The minimum number of doses given was 1, and the maximum was six over 48 h to maintain the SBP above 90 mm Hg. After resuscitation, patients were followed for 28 days. Analysis of patients’ data showed that trauma was the cause of hypovolemic shock in 29.41% of control and 47.06% of CQ, gastroenteritis in 44.12% of control, and 29.41% of CQ patients, however their demographics and baseline vitals in both groups were comparable. The shock index (SI) in CQ patients was significantly lower than control from 1 h (*p* = 0.0320) to 4 h (*p* = 0.0494) after resuscitation. After 3 days of resuscitation, significantly higher percentage (69.35%) of CQ patients than control patients (46.88%) had blood lactate level 1.5 mm or less. After 28 days, we observed significantly lower mortality (2.94%) in CQ group than control group (11.76%). CQ patients also showed an increase in PP and SBP. A significant increase in PP in the CQ group suggested improved stroke volume due to CQ resuscitation. A significantly greater number of patients had improved base deficit in CQ than the control group. Also, an 8.8% absolute reduction in 28-day all-cause mortality was observed in the CQ group along with improved acute respiratory distress syndrome (ARDS) and multiple organ dysfunction syndrome (MODS) scores (Gulati et al., 2019; Gulati et al., 2020; Gulati et al., 2021a; Gulati et al., 2021b). The meta-analysis of the mortality data obtained from our phase II and III studies found that mortality was 10.71% in the control group (*N* = 56) and 2.20% in the CQ group (*N* = 91) (*p* = 0.03), which indicated statistically significant reduction in mortality in CQ group (Gulati et al., 2021a). Based on these successful clinical trials Pharmazz Inc. has obtained marketing authorization to sell CQ as a drug (Lyfaquin^®^) for hypovolemic/hemorrhagic shock in India (Gulati et al., 2020; Gulati et al., 2021a), and a phase III IND application is approved by US FDA to conduct a trial on 430 patients.

Furthermore, we also explored the role of CQ in kidney damage protection because kidneys play an important role in blood pressure regulation and fluid homeostasis. Also, mortality in hemorrhagic shock patients is known to be linked to acute kidney failure (Harrois et al., 2018), and damaged kidney in shock leads to further disturbance in homeostasis and accelerates failures of other organs (Basile et al., 2012). We explored the role of CQ on kidney perfusion and the protection of renal tissues against hypoxic damage in a rat model of hemorrhage and acute kidney damage. We observed significantly improved kidney blood flow and decreased blood lactate levels in CQ-treated rats. Analysis of kidney tissues in these rats showed significant up-regulation (*p* = 0.024) of hypoxia-inducible factor 1a (HIF-1α) and reduced (*p* = 0.03) mitochondrial DNA damage than control rats. We also observed reduced acute kidney injury and apoptosis in kidneys in CQ rats (Ranjan et al., 2020; Ranjan et al., 2021). Thus, CQ, known to activate the α-adrenergic system, has proven to be an effective resuscitative agent for hypovolemic/hemorrhagic shock and potentially improves cardiovascular function, renal tissue perfusion, and protection. In the future, we will further explore the therapeutic potential of CQ in other types of shock, e.g., distributive shock such as septic shock and COVID-19, where the decompensated phase of shock is known to affect the cardiovascular system resulting in a state of hypovolemia as well as hypotension.

## 4. Conclusion

The common characteristics of the decompensatory phase of various types of shock include compromised cardiovascular activities with severe hypotension. Resuscitation with several vasopressors has demonstrated encouraging results, with an increase in vasocontraction and MAP at the initial phase of resuscitation. However, the complex sympathetic and parasympathetic signaling mediated through various ARs complicates the outcomes. Hence, for a better understanding of shock etiology and adrenergic receptor physiology, pharmacological interventions must be investigated for their efficacious use in the treatment and prevention of decompensated shock. Our group has addressed this issue by exploring the effect of α2 specific agonist, CQ, in hypovolemic/hemorrhagic shock. CQ activates α2B and α2A ARs, causing an increase in CO and a decrease in SVR with net results of increased blood pressure as well as tissue perfusion. It also helps in reducing blood lactate levels and improves survival. The clinical trials of CQ conducted in India demonstrated significantly improved survival, improved blood circulation, and reduced lactate level in shock patients compared to in-use standard resuscitative agents. Thus, α2 ARs could be suitable targets for treating or managing hypovolemic/hemorrhagic shock. Nonetheless, a deeper investigation of ARs and pharmacologic modulation of ARs using potent agonists like centhaquine (Lyfaquin^®^) are required to regulate the shock-induced decompensatory vasodilation and reestablish blood circulation, vascular volume and improve survival in different types of shock.
